# Early initiation and exclusive breastfeeding: Factors influencing the attitudes of mothers who gave birth in a baby-friendly hospital

**DOI:** 10.4274/tjod.90018

**Published:** 2017-03-15

**Authors:** Elif Yılmaz, Fatma Doğa Öcal, Zehra Vural Yılmaz, Meryem Ceyhan, Osman Fadıl Kara, Tuncay Küçüközkan

**Affiliations:** 1 Dr. Sami Ulus Maternity and Child Health and Diseases Training and Research Hospital, Clinic of Obstetrics and Gynecology, Ankara, Turkey; 2 Amasya University Faculty of Medicine, Department of Obstetrics and Gynecology, Amasya, Turkey

**Keywords:** Breastfeeding initiation, exclusive breastfeeding, complementary feeding

## Abstract

**Objective:**

To investigate the initation time of breastfeeding, exclusive breastfeeding rates, and complementary feeding practices during the first six months of life among mothers who gave birth in a baby-friendly hospital.

**Materials and Methods:**

A cross-sectional study was conducted with 350 mothers. Demographic characteristics, obstetric history and information about breastfeeding initiation were collected at the hospital. Information about factors affecting breastfeeding duration and feeding practices of the infants were obtained at the end of six months.

**Results:**

Some 97.4% of the mothers initiated breastfeeding, 60.1% within the first hour. Exclusive breastfeeding was maintained for six months in 38.9%. Low education levels of mother/father, random breastfeeding, rare breastfeeding at night, nipple problems, bottle/pacifier use, and lack of social support were found associated with early cessation. Planned pregnancy [odds ratio (OR=2.02)] and vaginal delivery (OR=0.3) were found as the most important factors in early initiation, whereas antepartum breastfeeding education (OR=7.17) was the most important factor for exclusive breastfeeding duration in the logistic analysis. More than half (61.1%) of the infants were partially/bottle fed for six months; the most common reason was the belief that breast milk was insufficient.

**Conclusion:**

Efforts to encourage mothers and society to breastfeed exclusively should be made as part of a primary public health strategy to prevent early cessation of breastfeeding.

## INTRODUCTION

The recommendation of the World Health Organization (WHO) is exclusive breastfeeding (EBF) of infants during the first six months of life, and breastfeeding (BF) for two years or beyond with complementary foods for achieving optimal growth and health^(1)^. In addition to providing essential nutrients to infants, breastmilk has been shown to be related to decreased sudden infant death syndrome, respiratory-gastrointestinal tract infections, and allergic diseases, as well as a lower risk of developing obesity, cardiovascular disease, diabetes, and hematologic malignancies in future life^(2,3,4)^. Reduction in postpartum blood loss due to increased uterine activity and greater weight loss compared with mothers who bottle feed are the reported benefits of BF for mothers^(2,3)^.

Despite all recommendations of the WHO, the rates of initiation and duration of BF are still far from expectations worldwide. According to the United Nations Children’s Fund (UNICEF), EBF rates for the first 6 months have not changed significantly since 1990 and are around 36%^(5)^.

Various factors have been shown associated with both the initiation and duration of BF. It has been shown that advanced age, higher maternal educational level, higher socioeconomic status, BF education, social support, and an infant with birthweight over 3 kg and >38 weeks gestation have a positive affect on BF^(6,7,8,9,10,11)^. The belief of mothers that their milk is inadequate, the failure to provide adequate information and support from healthcare workers, breast problems due to incorrect BF, and the increased use of bottle-feeding/pacifiers are important causes for early discontinuation of BF^(7,12,13,14)^.

According to the Turkish Population and Health Survey (TPHS) 2013 report, BF is very common in Turkey, with 96% of children breastfed for some period. However, despite many encouraging studies condected in our country, both early initiation and exclusive BF rates are still far below the desired levels. Only 50% of childen are breastfed within the first hour and the median duration of EBF is only 1.2 months. The rate of EBF during the first six months fell to 30.1% in 2013, which was 41.6% in 2008^(15,16)^.

The main objective of the present study was to determine the initation time of BF and complementary feeding practices during the first six months of life among Turkish mothers who gave birth in a baby-friendly hospital, risk factors associated with lack of early initiation, and EBF.

## MATERIALS AND METHODS

A cross-sectional study was performed in the maternity ward of a tertiary hospital, one of the major maternity centers in Ankara, the capital of Turkey, between March and October 2015. A total of 350 mothers aged ≥20 years who gave birth in the hospital participated in the study on a voluntary basis. The exclusion criteria were multiple pregnancies, preterm births (<37 weeks), foreign patients, women with health problems, babies with health problems, and those who required the neonatal intensive care unit or intubation. The study was approved by the Keçiören Training and Research Hospital Local Ethics Committee (approval no: 2012-KAEK-15/1073) and was performed in accordance with the ethical standards described in an appropriate version of the 1975 Declaration of Helsinki, as revised in 2000.

Our hospital is a certificated baby-friendly institution and all mothers are explained the benefits of breastmilk and given BF education after delivery by lactation consultants.

After being informed about the study, informed written consent was obtained from the mothers. A questionnaire consisting of questions about demographic characteristics, obstetric history, and BF history was completed by two research assistants 12-24 hours after birth. The survey also investigated whether the infant received prelacteal feeds and their nature. Information about gestational age, birth weight, sex of the infant, and type of birth were obtained from hospital records. When discharging from the hospital, all patients were informed that they would be called by the research assistant at the end of the sixth month postpartum in order to get the infant’s nutritional information and feeding practices. During this call, information about BF, factors affecting BF and its duration, and feeding practices of the infant were obtained.

Initiation time and related factors were assessed for over 350 patients. Assessments related to BF duration and EBF were peformed with 329 patients, excluding the 21 patients that could not be reached at the end of six months.

### Definitions

Definitions included in the study:

-Early breasfeeding was defined as BF within one hour postpartum.

-Initiation time of first breastfeed was the time at which the baby was first breastfed after delivery.

-Exclusive breastfeeding was defined as BF only since birth and no other supplemental fluids.

-Partial breastfeeding was defined as mainly BF combined with supplemental foods.

-Bottle-feeding was defined as only feeding with formula or non-human milk without BF.

-Complementary feeding was defined as transition from EBF to family foods.

### Statistical Analysis

The analyses were performed using Statistical Package for the Social Sciences software version 15.0 (SPSS Inc., Chicago, IL, USA). Results are presented as mean ± standard deviation and n (%). The suitability of continuous variables to normal distribution was analyzed using the Kolmogorov-Smirnov test. Nominal data were expressed using cross tables depending on initiation times before or after one hour, and EBF more or less than six months. Differences between groups were assessed using χ^2^, Fisher or Yates tests for categorical variables. Multivariate logistic regression analysis was used to determine which factor best predicted BF within one hour postpartum, and which factor predicted EBF for six months. The Hosmer-Lemeshow test was used to determine the goodness of fit of the logistic regression model. A p value of <0.05 was considered statistically significant.

## RESULTS

### Socio-demographic and obstetric characteristics

The mean age of the study group at birth was 27.58±5.4 years (range, 20-42 years), and the mean gestational age was 38.7±1.2 weeks (range, 37-41 weeks). The majority of the mothers were housewives, 11.7% were working mothers, and 68.4% were with their babies at home during the first six months. Regarding education, 64% of the mothers were educated at primary school or illiterate. Just under half (47.7%) of the women experienced a vaginal delivery and 4% of the babies weighed <2500 gr. The mean birthweight of babies was 3234.34±436.1 g (range, 2080-4800 g), and 55.1% of the babies were girls.

### Breastfeeding initiation and influencing factors

In total, 97.4% of the mothers in this study initiated BF. Of the 341 patients, 60.1% initiated BF within the first hour, and 22.6% within the second hour after birth. Of the mothers who gave vaginal birth, 76.8% initiated BF within the first hour, whereas this ratio was 44.6% in the cesarean group. The mean first BF time was 1.67±1.0 hours postpartum (range, 1-6 hours), 1.33±0.7 hours for vaginal delivery, and 1.97±1.1 hours in the cesarean group.

Table 1 shows the characteristics of the study group according to the BF initiation time. Planned pregnancy and vaginal delivery were found as factors that had an affect on the initiation time of BF.

Planned pregnancy and vaginal delivery were found as significant factors for early BF in the logistic regression analysis (Table 2). Early BF was significantly higher in the vaginal delivery group.

### Breastfeeding patterns, rates and infant feeding practices of the study group

Bottle feeding from birth was reported in 2.7% of the mothers, 88.3% of the mothers stated that breast milk was the first food taken by the newborns, the rest of the newborns were fed with other fluids before breast milk and then breastfed. The number of mothers who stated that they stopped BF from the first to fifth months were 12, 15, 29, 30, and 54 mothers, respectively.

Partial bottle feeding was disclosed by 61.1% of the mothers, and 38.9% exclusively breastfed until six months from birth (Table 3).

When the partial feeding group was asked why they were giving formula/other drinks, the most common responses were concerning the insufficiency of breastmilk (39.6%), a family belief that the baby’s weight gain was inadequate (25.5%), supplementing BF (21.9%), and convenience of the mother (13%). EBF rates fell whereas partial/bottle feeding rates increased as babies grew older (Table 4).

Some 39.8% of the infants in the study group were introduced to solid foods before 6 months, which is the recommended age by the WHO. Solid foods were more commonly initiated around 4-6 months (6.9% at 4 months, 12.8% at 5 months, 20.1% at 6 months, and 60.2% at the end of six months). The mean infant age at which solid foods were introduced was 5.33±0.9 months and for formula milk it was 2.23±1.8 months.

### Exclusive breastfeeding duration and influencing factors

EBF for 6 months was maintained by 38.9% of the mothers. The mean duration of BF was 4.86±1.6 months, and the mean duration of EBF was 3.66±2.3. Table 5 summarizes the maternal and infant characteristics of the EBF group compared with the early cessation groups. Low education levels of mother and father, not receiving antepartum BF education, random BF, rare BF at night, nipple problems, bottle/pacifier use, and lack of social support were found as variables associated with early cessation of BF. In the multivarite analysis, antepartum BF education was found as the most significant factor in EBF duration (Table 2).

## DISCUSSION

This study presents the BF and complementary feeding practices of Turkish mothers, with a focus on risk factors associated with lack of early initiation and EBF. Planned pregnancy and vaginal delivery were found as the most important factors in early initiation, whereas antepartum BF education was the most important factor for EBF duration in logistic analysis. Education level of mother and father, frequency of BF, number of BFs at night, nipple problems, bottle/pacifier use, and social support were found as other factors that had statistically significant effects on the duration times of EBF. 

In our study, the percentage of early initiation was 60.1%. This ratio can be considered in the good group according to the classification of WHO [poor (0-29%), fair (30-49%), good (50-89%), and very good (90-100%)]^(17)^. Although this result is not at the desired level, it is better than the initiation times found in other studies conducted in our country, which determined rates between 9.9%-50%^(16,18,19)^. Being a baby-friendly hospital and providing all mothers with lactation consultancy after birth may have had an effect on these results. Despite the efforts on BF education all over the country, the premature introduction of other fluids before BF (11.2%) is still common because of a superstition among people.

One of the most important factors found to affect early initiation in our study was delivery type, consistent with the literature. Mothers who gave birth vaginally were significantly more likely to initiate early BF compared with cesarean deliveries. As was shown by several studies, cesarean birth is one of the most important obstacles that causes a delay in the initiation times of BF^(18,20,21)^. Pain after surgery, significant discomfort in holding and positioning the baby, delayed skin-to-skin contact, delay in the production of breastmilk, limited mobility, and needing extra help for BF could account for this delay. Women who undergo cesarean delivery may need additional help to attain comfortable and correct positioning of their infant for BF. In recent years, cesarean section rates are above the expected levels around the world. According to Turkey Health Statistics, 50% of all births were performed by cesarean section in 2013, similar to our results (52.3%)^(22)^. Developing policies to reduce cesarean rates is essential, but it should also be noted that providing special lactation consultancy and emphasizing skin-to-skin contact in patients where cesarean is mandatory is necessary.

In the study group, the initiation times of BF in women who gave birth after a planned pregnancy were significantly less than for women with unplanned pregnancies. Consistent with our results, Taylor and Cabral^(23)^ found a stronge inverse association between unwanted pregnancies and BF in their study of 6733 first singleton live births. Kost et al.^(24)^ reported that women with unwanted pregnancies were less likely to breastfeed their babies than those who intended to conceive. Therefore, as a woman’s attitude toward her baby can affect her likelihood of baby-care and consequently her decision to breastfeed, the importance of lactation consultancy should be kept in mind for women with unplanned pregnancies.

The majority of mothers in the study group initiated BF, but only 38.9% of them exclusively breastfed their infants for six months. Although this result is better than the 30.1% detected in the TPHS 2013 report, it is still far behind the desired levels. According to UNICEF, global rates of BF have remained stagnant since 1990, with only 36% of children aged less than six months were exclusively breastfed in 2012, worldwide^(5)^. The higher results of our study compared with the TPHS and UNICEF may be explained by the fact that our hospital is in a semi-urban region that serves a relatively better income group who have higher antepartum follow-up and BF education rates compared with other regions of our country. Also, our hospital is a baby-friendly hospital that aims to promote and support BF, and this might have had an influence on the results. It should be kept in mind that there still exists a need for encouraging mothers to exclusively feed their babies with breast milk for up to six months. Mothers should be informed about the benefits of breast milk, BF techniques, and how to avoid circumstances that would negatively affect this.

Both maternal and paternal education were found as effective factors for BF duration in our study. Similar to our results, low maternal and paternal education were found as risk factors for early weaning of BF in the literature^(6,10)^. Maternal working status has been previously negatively linked to BF, EBF in housewives was reported more than in working mothers^(12,20)^. Interestingly, this finding is in contrast with our results. The higher EBF rates in the working mothers in our study group may be associated with their late return to work and being at home, especially during the first six months.

In our study group, mothers who breastfed more frequently during the day and the night, as suggested by our health staff, breastfed their infants significantly longer than did mothers who breastfed their infants upon demand. It is known that maternal milk production is positively correlated with the demand of the infant and frequent feeding maintains breast milk supply^(1,25)^. The findings of our study also support this information.

Bottle/pacifier use and nipple problems were reported as negative influencing variables of BF duration^(26^). It is known that the use of bottle/pacifier possibly changes the baby’s oral dynamics and sucking pattern and may lead to both a reduction in BF frequency and breast demand. For improving EBF, effective strategies to reduce bottle/pacifier should be developed for use among infants, especially those younger than 6 months. Nipple problems are common among BF mothers. Lack of information about BF positions and poor latch of the baby are the most frequent cause of damage to nipples^(27)^. As these problems are the common causes of early weaning of BF, health staff should pay special attention to mother’s breasts in the early postpartum period. Proper BF education by a lactation consultant is essential in the prevention of these problems and ensuring the continuity of BF.

Regression analysis indicated that antepartum BF education was the most significant predictor for the duration of BF. One of the top reasons for the early cessation of BF is mothers’ inadequate knowledge about the importance of breast milk and BF techniques. Successful BF starts when the mother thinks she is going to breastfeed her baby and believes that she can accomplish it. The affect of antepartum BF education on increasing EBF rates has been shown in several studies^(28,29)^. In a study performed in Israel to explore the effect of BF education given in the perinatal period, it was found that both the initiation and the duration rates of BF were increased^(30)^. Therefore, antepartum education should be considered by health staff to increase the self-confidence of mothers.

In the present study, the most commonly reported reason for starting supplementary drinks was the belief of breastmilk insufficiency. The concern about milk insufficiency among mothers is still very common and stems from having insufficient information on the proper techniques to increase breast milk. Mothers should be relaxed so that they are able to produce enough breastmilk for the proper growth of their infants, and informed about the importance of frequent feeding with the correct technique for stimulating optimal milk production^(1,25)^.

Similar to our study, results of early introduction of complementary foods, contrary to WHO recommendations are of great concern, especially in developing countries. Mothers should be warned about the impact of premature introduction of complementary foods on early termination of EBF, without conferring any growth advantage over EBF^(20)^.

### Study Limitations

There are several limitations to our study. The nature of data collection was retrospective and the responses were self-limited, which may have led to over or under estimation of BF practices and duration. To minimize these errors, all the questionnaires were completed by the same author to ensure consistent technique. Enough time was allocated to all patients to avoid rushing and insufficient thinking time of the patients for the questions. Also, the cross-sectional nature of the study prevented determining causal relationships of knowledge, attitudes, or interest with the rates of BF. However, the hospital where the study was conducted is a large women’s health center located in a semi-urban region, which exhibits a close profile to the entire country. At the same time, the study is important in indicating the effectiveness of baby-friendly hospital practices, especially postpartum BF education. Larger prospective studies are needed to clarify BF duration rates, factors that affect these practices, and practical things that should be done to increase these rates in our country.

## CONCLUSION

In conclusion, this study reported important factors that affect the initiation and the duration of BF. Despite all the efforts spent on this subject, both the initiation and duration of BF rates are still below the desired levels in our country. The results of our study indicate the importance of awareness of both mothers and their family members regarding the significance and benefits of BF. Efforts should therefore be made to ensure both professional and social support to mothers to prevent early cessation of BF.

## Figures and Tables

**Table 1:**
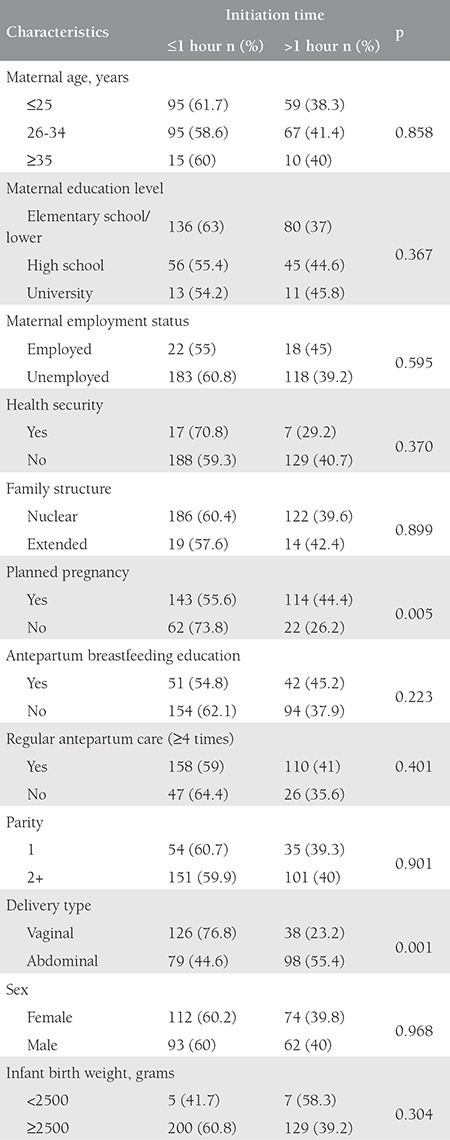
General characteristics of the study group according to the initiation time

**Table 2:**
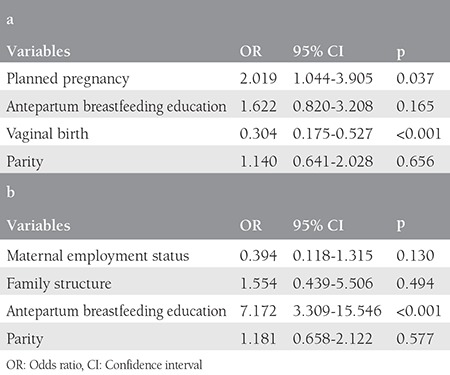
Logistic regression analyses for factors associated with initiation time of breastfeeding (a) and with exclusive/predominantly breastfeeding (b)

**Table 3:**
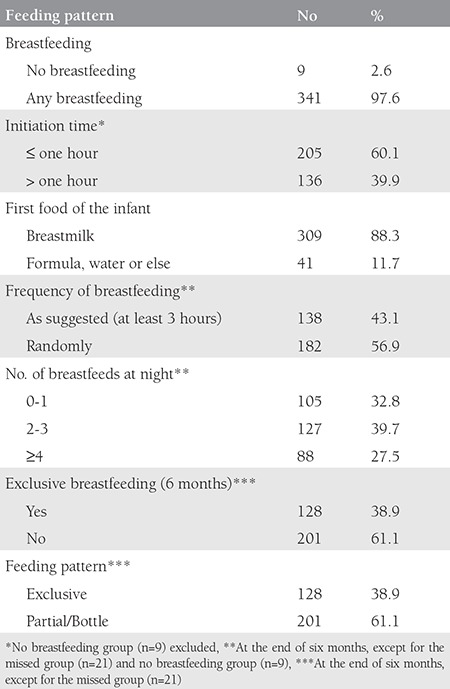
Breastfeeding, feeding patterns, and breastfeeding rates of the study group

**Table 4:**
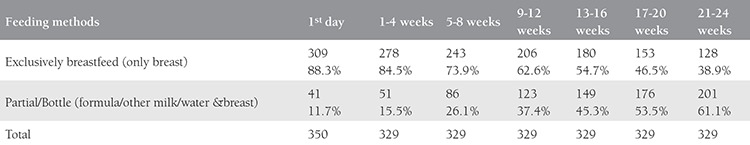
Infant feeding patterns during the first six months

**Table 5:**
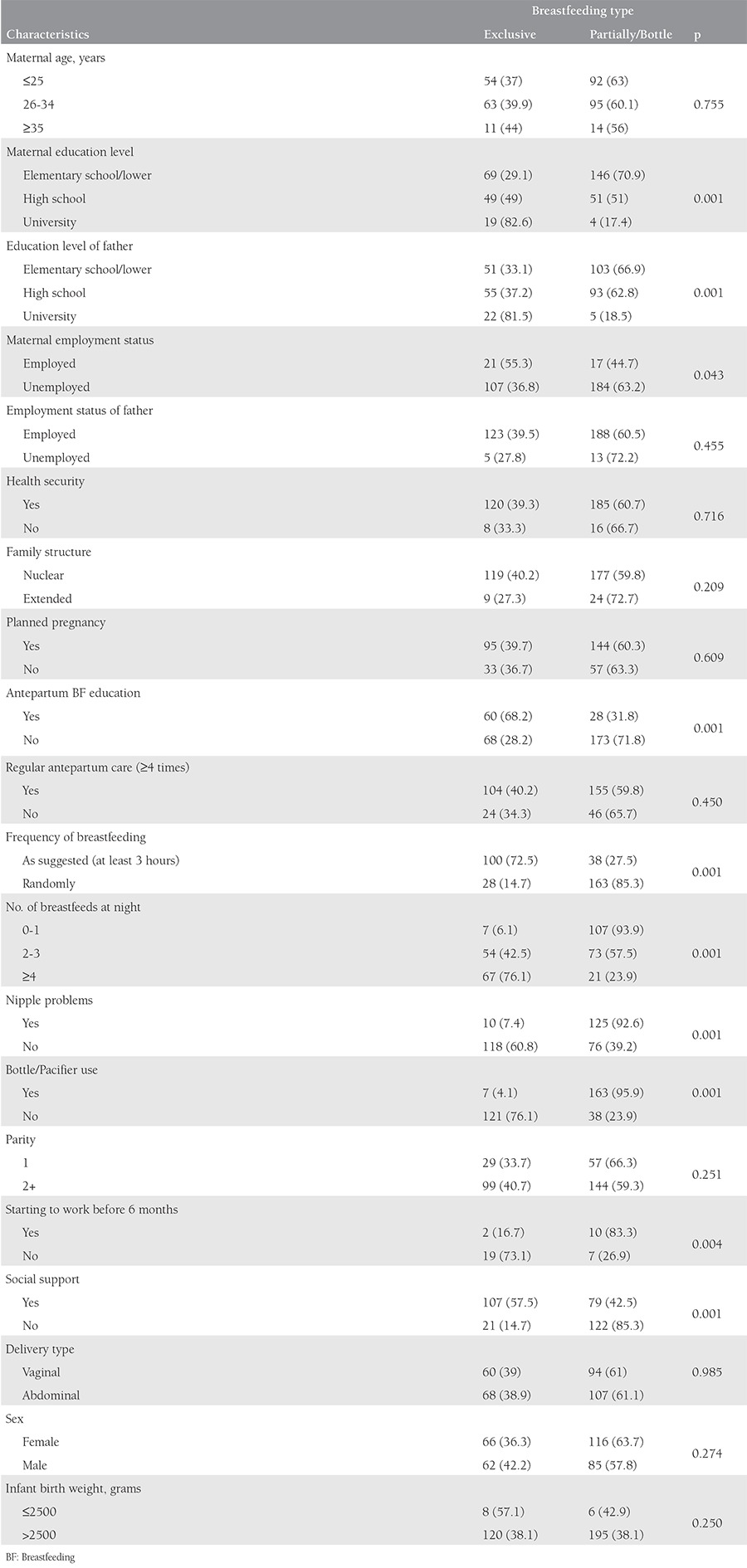
General characteristics of the study group according to breastfeeding type
